# Serum osteocalcin level and its association with carotid atherosclerosis in patients with type 2 diabetes

**DOI:** 10.1186/1475-2840-12-22

**Published:** 2013-01-23

**Authors:** Li Sheng, Wenjie Cao, Bingbing Cha, Zhaoping Chen, Fang Wang, Jun Liu

**Affiliations:** 1 Department of Endocrinology, Shanghai Fifth People’s Hospital, Fudan University, 801 Heqing Road, Shanghai, China; 2 Department of Neurology, Huashan hospital, Shanghai medical college, Fudan University, 12 Middle Wulumuqi Road, Shanghai, China

**Keywords:** Osteocalcin, Intima-media thickness, Carotid atherosclerotic plaques, Atherosclerosis

## Abstract

**Objective:**

To investigate the association of serum osteocalcin with carotid atherosclerosis in patients with type 2 diabetes.

**Methods:**

We performed a cross-sectional community-based study in metropolitan area. Serum total osteocalcin was measured by radioimmunoassay in 382 men and 435 postmenopausal women. The carotid artery intima-media thickness (IMT) and carotid plaques (PLQ) were measured by B-mode ultrasound.

**Results:**

The crude mean of serum osteocalcin concentrations were 4.52±2.43 ng/ml for men and 5.75±2.92 ng/ml for postmenopausal women (P <0.001), respectively. Osteocalcin levels were associated inversely with age, fasting serum insulin, HOMA-IR, ALT, triglycerides, total cholesterol, LDL- cholesterol, CRP (all *P*<0.001) and positively with adiponectin and HOMA-B (all P<0.05). After multiple adjustment, the odds ratios (ORs) were substantially higher risk for carotid plaques (OR 1.77 for 1 SD decrease in osteocalcin, 95% CI 1.23-2.76, p=0.005). These associations remained significant after further adjustment for potential confounder.

**Conclusions:**

Serum osteocalcin levels is an independent risk factor for carotid atherosclerosis in patients with type 2 diabetes.

## 

The increased cardiovascular risk in type 2 diabetes mellitus is reflected by atherosclerosis, which is an established predictor of coronary heart disease and stroke in older subjects [1-4]. This diabetes-associated increase in atherosclerosis is associated with conventional cardiovascular risk factors, such as central obesity, high blood pressure, and dyslipidaemia, as well as with insulin resistance [5,6].

Osteocalcin is a 49-amino acid bone matrix noncollagen protein expressed mainly by osteoblasts [7]. It is a specific biochemical marker of bone turnover and bone formation involved in bone mineralization and calcium homeostasis [8]. Studies have verified that adipose tissue could regulate bone remodeling through the adipokine leptin by acting on osteoblasts [9,10]. In turn, bone modulates energy metabolism in a feedback loop. The novel function for the skeleton unraveled its importance as an endocrine organ. A bone-derived protein, osteocalcin, has raised much attention as a hormone regulating glucose metabolism and fat mass. Recently, osteocalcin has been recognized as a bone-derived hormone to regulate energy metabolism. Osteocalcin knockout mice exhibited glucose intolerance, increased fat mass, insulin resistance, decreased expression of insulin target genes in liver and muscle, and decreased adiponectin gene expression in adipose [11], while administration of recombinant osteocalcin increased insulin secretion, decreased blood glycaemia and weaken the development of obesity [12].

Previous work has demonstrated that osteocalcin levels are inversely associated with glucose and total adiponectin levels, fat mass, and atherosclerosis parameters in patients with type 2 diabetes [13]. Furthermore, Chen et al. observed that serum osteocalcin levels of participants with self-reported CVD were significantly lower than those without in middle-aged and elderly Chinese [14]. Recently, a cohort study has demonstrated that serum osteocalcin is a strong determinant of severity of coronary atherosclerosis in Chinese men [15].

In keeping with these observations, it is plausible to consider serum osteocalcin as a promising candidate for risk assessment and a potential intervention target for diabetic macrovascular diseases. To better evaluate the possible role of serum osteocalcin in the development of diabetic macrovascular diseases, we examined the association of serum osteocalcin levels and development of carotid atherosclerosis in type 2 diabetes.

## Methods

### Study sample

A cross-sectional study to evaluate the prevalence of diabetic complications in Chinese patients diagnosed with type 2 diabetes aged over 50 was planned in the Shanghai downtown residential areas administered by 10 residents’ committees were cluster sampled in Shanghai central area. Questionnaires to identify diabetes history were sent to every household in the 10 residential areas and collected by primary care clinicians and endocrinologists. The 902 Chinese patients diagnosed with type 2 diabetes were identified by questionnaire. The criteria of diagnosis of type 2 diabetes recommended by ADA in 1997 were adopted. Finally, 817 (90.6%) (382 men and 435 postmenopausal women)Chinese patients diagnosed with type 2 diabetes were enrolled in our study. Written consent was obtained from all participants. The study was approved by the Institutional Review Broad of Shanghai Fifth People’s Hospital, Fudan University School of Medicine.

### Data collection

A standardized questionnaire was used by trained physicians to collect information such as age, sex, smoking (yes/no), alcohol drinking (yes/no) and self-reported history of diabetes, hypertension, dyslipidaemia, coronary heart disease and stroke.

All subjects were assessed after overnight fasting for at least 10 h. Anthropometric measurements including height, weight, waist circumference, hip circumference and blood pressure were performed by trained physicians. Body mass index was calculated as the weight in kilograms divided by the square of height in metres.

### Biochemical measurements

Peripheral venous blood samples were collected. Fasting plasma glucose, total cholesterol, triglycerides, low-density lipoprotein cholesterol (LDL-c), high-density lipoprotein cholesterol (HDL-c), serum creatinine, blood urea and serum uric acid were measured on an automatic analyzer (Hitachi 7080; Tokyo, Japan). Glycated haemoglobin was tested by high-pressure liquid chromatography (HLC-723G7, Tosoh, Shanghai, China).

### Carotid ultrasonography

Trained technicians performed B mode ultrasonography using an Acuson Sequoia 512. Images were stored and read centrally at Shanghai Fifth People’s Hospital Image Center. The ultrasound scanning protocol in our study was modified in terms of procedures used in previous studies [16,17]. Computer-assisted edge-detection software was not used for measurement of carotid IMT. A lateral view of bilateral images of common carotid arteries (1 cm proximal to the dilatation of the carotid bulb), carotid bulb (identified by the loss of the parallel wall present in the common carotid artery) and internal carotid artery (1 cm distal to the tip of the flow divider that separates the external and internal carotid arteries) was obtained. Sonographers recorded the images and completed ultrasound readings. Intima-media thickness is the distance between the lumen-intima interface and the media-adventitia interface [18]. Common carotid artery IMT was defined as the mean of the maximum IMT in both right and left sides of common carotid artery. Reproducibility was high, with small coefficients of variation (CV) value of measurement of IMT (3.5%).

The plaque of carotid artery (common carotid artery, carotid bulb, and internal carotid artery) is defined as a localized protrusion of the internal part of the vessel wall into the lumen of 50% of the surrounding IMT value. Plaque presence was defined as ≥1 plaque in any of the carotid arteries [19].

### Measurements of serum C-reactive protein (CRP) and total osteocalcin

The serum CRP were determined in duplicate by ELISA with Duoset kit (DY1707; R&D Systems, Minneapolis, MN) as recommended by the manufacturer. The ELISA system had an intraassay coefficient of variation of 3.5-9.9% and an interassay coefficient of variation of 4.1-10.3%, respectively. Serum total osteocalcin was measured by radioimmunoassay (Atom-hitech Beijing, China) with intra and interassay coefficients of variation of 2.4–8.7and 2.5–9.5% respectively.

### Statistical analysis

Normally distributed data were expressed as means ± SD, whereas variables with a skewed distribution were reported as median (interquartile range) and log transformed to approximate normality before analysis. Categorical variables were represented by frequency and percentage. Correlation coefficients between osteocalcin and metabolic features were calculated by partial correlation analysis. Multivariate linear regression models were used to estimate the determinants of IMT. Potential confounding variables including age, gender, smoking, duration of diabetes, BMI, waist circumference, systolic blood pressure, diastolic blood pressure, CRP, fasting plasma glucose, serum creatinine, serum urea, serum cholesterol, LDL-c and HDL-c were controlled in the regression models. The interaction of confounding variables was assessed. Multivariate logistic regression models were used to estimate the association of carotid atherosclerotic plaques (PLQ) with osteocalcin. Potential confounding variables including age, gender, smoking, alcohol drinking, self-reported CVD, family history of diabetes, eGFR, HbA1c, CRP, waist circumference, omeostatic model assessment of insulin resistance (HOMA-IR), waist circumference, and body mass index (BMI) were controlled in the regression models. We defined subjects with no carotid atherosclerotic plaques as 0 (n =352), and carotid atherosclerotic plaques as 1 (n =465). All statistical analysis was performed with the SPSS Statistical Package (version 13.0; SPSS Inc., Chicago, IL). A p value of less than 0.05 was considered to be statistically significant.

## Results

### General characteristics of participants

In all, 817 participants were analyzed, among which 382 were male, 435 were female (Table 1). The average age was 61.46±11.58 years. The average duration of type 2 diabetes was 7.53±7.29 years. Serum triglycerides and CRP were skew-distributed and had been log transformed to approximate normality before analysis. Compare to female subjects, the male subjects exhibited higher levels of waist circumference (*P* < 0.05), waist-to-hip ratio (*P* < 0.05), serum creatinine (*P* < 0.05), serum urea (*P* < 0.05), serum uric acid (*P* < 0.05) and CRP (*P* < 0.05), but lower levels of BMI (*P* < 0.05), adiponectin (*P* < 0.05), serum cholesterol (*P* < 0.05), LDL-c (*P* < 0.05) and HDL-c (*P* < 0.05).

**Table 1:** General characteristics of type 2 diabetic patients [Data were reported in mean ±SD or median (interquartile range)]

	**Male**	**Female**
*n*	382	435
proportion of PLQ (%)	61.5%	52.9%
Age (years)	61.28±11.74	61.60±11.82
duration of diabetes (years)	7.51±7.29	7.57±7.61
BMI (kg/m^2^)	24.87±2.78	25.62±3.67*
Waist circumference (cm)	86.75±8.39	82.87±8.91*
Waist-to-hip ratio	0.90±0.08	0.87±0.08*
DBP (mmHg)	82.27±11.42	81.45±11.39
SBP (mmHg)	138.47±19.62	139.75±21.39
FPG (mmol/L)	8.52±3.31	8.48±3.27
Fasting serum insulin (μU/ml)	6.78 (4.62-10.53)	6.85 (4.59-10.13)
HbA1c (%)	7.19±1.66	7.14±1.49
Serum uric acid (μmol/L)	315.47±83.29	281.28±69.74*
Serum urea (mmol/L)	6.46±1.91	5.91±1.62*
Serum creatinine (μmol/L)	80.23±24.54	60.68±16.76*
Serum cholesterol (mmol/L)	5.11±1.13	5.65±1.12*
^#^Serum triglycerides (mmol/L)	1.53 (1.07-2.22)	1.64 (1.15-2.41)
LDL-c (mmol/L)	2.92±0.84	3.122±0.95*
HDL-c (mmol/L)	1.24±0.40	1.38±0.38*
^#^CRP (mg/l)	2.40 (0.89-5.21)	1.75 (0.82-5.51)*

Abbreviations: DBP diastolic blood pressure, SBP systolic blood pressure, FPG fasting plasma glucose, LDL-c low-density lipoprotein cholesterol, HDL-c high-density lipoprotein cholesterol.

* *P* < 0.05.

^#^Serum fasting insulin, triglycerides and CRP were skew-distributed and had been log transformed to approximate normality before analysis.

### Correlation analysis of the relationship between osteocalcin and clinical variables

In spearman analysis, serum osteocalcin correlated with age (r =−0.113, <0.001), CRP (r =−0.073, P=0.031), fasting plasma glucose (r =−0.085, P<0.001), fasting plasma insulin (r =−0.155, P<0.001), HOMA-IR (r =−0.145, P<0.001), total cholesterol (r =−0.133, P<0.001), triglycerides (r =−0.102, P<0.001), and LDL-c (r =−0.087, P=0.014). However, serum osteocalcin was not associated with BMI, duration of diabetes, waist circumference, waist-to-hip ratio, systolic blood pressure, diastolic blood pressure, HbA1c and HDL-c (all P> 0.05) Table 2.

**Table 2:** **Correlation between serum osteocalcin and other parameters in patients with type 2 diabetes**^**a**^

**Variable**	**Correlation coefficient**	**P value**
Age	−0.113	<0.001
BMI	−0.062	0.117
Duration of diabetes	0.037	0.425
Waist circumference	0.032	0.479
Waist to hip ratio	−0.057	0.082
SBP	0.042	0.312
DBP	−0.035	0.457
CRP	−0.073	0.031
FPG	−0.085	0.025
Fasting plasma insulin	−0.155	<0.001
HbA1c	−0.023	0.573
HOMA-IR	−0.145	<0.001
TC	−0.133	<0.001
TG	−0.102	<0.001
LDL-c	−0.087	0.014
HDL-c	0.051	0.074

SBP, systolic blood pressure; DBP, diastolic blood pressure; TG, triglycerides; TC, total cholesterol; LDL-c, LDL cholesterol; HDL-C, HDL cholesterol.

^a^All correlation coefficients were calculated after adjustment for age, gender.

### Association between serum osteocalcin and IMT

Partial correlation analysis demonstrated that serum osteocalcin correlated with carotid IMT in diabetic patients after adjusting for age and gender (r =−0.110, P=0.005). After adjusting for age, gender HbA1c and HOMA-IR, osteocalcin still correlated with carotid IMT (r =−0.083, P=0.038). This correlation remained (r =−0.080, *P*=0.043) even after adjustment for age, gender HbA1c, HOMA-IR and serum CRP.

In the multivariate linear regression model, carotid IMT was set as dependent variable, while age, gender, smoking, alcohol drinking, duration of diabetes, BMI, waist circumference, systolic blood pressure, diastolic blood pressure, serum osteocalcin, HOMA-IR, CRP, fasting plasma glucose, serum creatinine, serum urea, serum cholesterol, triglyceride, HDL-c and LDL-c were set as independent variables. We tested the interactions of gender and CRP, gender and TC, gender and TG, gender and LDL-c as well as gender and HDL-c, but we failed to find any of significance. The multivariate linear regression analysis demonstrated that age, gender, systolic blood pressure, serum osteocalcin, LDL-c and HDL-c were independently associated with carotid IMT (Table 3).

**Table 3:** Multivariate linear regression analysis: independent predictors of carotid artery IMT among 817 type 2 diabetic patients

**Dependent variable**	**Independent variables**	**Standardized coefficients**	***P***	**Partial *r***
Carotid IMT	Age	0.312	2.5×10^-7^	0.312
	Sex	−0.152	2.14×10^-2^	−0.145
	SBP	0.159	9.84×10^-3^	0.168
	Osteocalcin	−0.181	4.95×10^-3^	−0.187
	LDL-c	0.142	2.51×10^-2^	0.148
	HDL-c	−0.194	3.95×10^-3^	−0.189

The abbreviations are the same as in Table 1.

### Association between serum osteocalcin and carotid atherosclerotic plaques

As presented in Table 4, reduced osteocalcin was associated with increased risk of carotid atherosclerotic plaques. Decreased serum osteocalcin indicated high risk for carotid atherosclerotic plaques (OR 1.77 for 1 SD decrease in osteocalcin, 95% CI 1.23-2.76, p=0.005), after adjustment for age, gender, smoking, alcohol drinking, duration of diabetes, BMI, waist circumference, systolic blood pressure, diastolic blood pressure, serum osteocalcin, HOMA-IR, CRP, fasting plasma glucose, serum creatinine, serum urea, serum cholesterol, triglyceride, HDL-c and LDL-c.

**Table 4:** The risk of carotid atherosclerotic plaques associated with 1 SD decrease in osteocalcin

	**Model**	**PLQ**	**p value**^**a**^
Total	Model 1	1.93 (1.32-3.01)	<0.001
	Model 2	1.86 (1.31-2.95)	<0.001
	Model 3	1.82 (1.28-2.94)	<0.001
	Model 4	1.81(1.24-2.82)	<0.001
	Model 5	1.76 (1.22-2.77)	0.005
men	Model 1	2.13 (1.41-3.69)	<0.001
	Model 2	2.02 (1.50-3.57)	<0.001
	Model 3	1.91 (1.32-2.88)	<0.001
	Model 4	1.83(1.27-2.85)	<0.001
	Model 5	1.81 (1.25-2.83)	<0.001
women	Model 1	1.88 (1.29-3.12)	<0.001
	Model 2	1.84 (1.27-2.99)	<0.001
	Model 3	1.80 (1.24-2.98)	<0.001
	Model 4	1.79(1.22-2.92)	<0.001
	Model 5	1.75 (1.19-2.85)	0.008

Values are ORs (95% CI).

Model 1: Unadjusted. Model 2: Adjusted for age, sex, smoking (yes/no), alcohol drinking (yes/no), family history of diabetes (yes/no). Model 3: Further adjusted for waist circumference, HOMA-IR, BMI and waist/hip ratio. Model 4: Further adjusted for serum triacylglycerol, total cholesterol, HDL- and LDL-cholesterol. Model 5: Further adjusted for inflammatory factors (CRP) and treatments (including antihypertensive therapy, antihyperlipidemic therapy and antihyperglycemic therapy). aFor the risk of PLQ, we defined subjects with no carotid atherosclerotic plaques as 0, and carotid atherosclerotic plaques as 1 from the analysis.

## Discussion

In this study, we found that serum osteocalcin levels showed association with the risk of carotid atherosclerosis from the cross-sectional data in type 2 diabetes. Moreover, these associations are independent of lifestyle factors, duration of diabetes, family history of diabetes, and, remarkably, CRP, HbA1c, HOMA-IR and BMI.

Serum osteocalcin correlated negatively with TG and TC in our study, which is consistent with previous studies. It is well established that elevated serum TC was an independent risk factor for the development of CVD. Given that serum osteocalcin levels were significantly associated with CRP and TC levels, it is plausible to consider osteocalcin as a promising candidate for risk assessment and a potential intervention target for CVD. In a Japanese study, serum osteocalcin was negatively associated with intima-media thickness of common carotid artery in type 2 diabetic men [13]. In another study, serum levels of osteocalcin were inversely associated with the metabolic syndrome and the severity of coronary artery disease in Chinese men [15]. Moreover, an Australian study showed that serum osteocalcin levels predicted all-cause and CVD-related mortality in community-dwelling older men [20]. Interestingly, in line with our hypothesis, we observed that serum osteocalcin levels is an independent risk factor for carotid atherosclerosis in patients with type 2 diabetes. Certainly, prospective studies with solid clinical end points are urgently needed to clarify whether low osteocalcin level plays a causal role in the development of atherosclerosis.

Endothelial inflammation plays a major role in the development of cardiovascular disease (CVD) [21]. The inflammatory process is complex, with various cellular mediators known to contribute [22]. We further tested the correlation between chronic low-grade inflammation and osteocalcin. In our study, serum osteocalcin correlated negatively with CRP. CRP is a pattern-recognition molecule of innate immunity as an acute-phase reactant and a hallmark of low-grade systemic inflammation [23,24]. CRP is a well known serum marker of chronic low-grade inflammation and has been associated with diabetes, hypertension and CVDs. Hence, the results of current study might be partially mediated via the mechanism of chronic low-grade inflammation.

Moreover, there is evidence to show the influence of bone proteins on cardiovascular disease [25-33]. During atherogenesis, bone matrix proteins, including osteocalcin, may have a regulatory role in the atherosclerotic calcification process [34]. Recent evidence suggests that osteoblast-like cells are present in the vasculature and capable of calcifying vascular cells. Furthermore, paracrine regulators of bone metabolism such as osteocalcin, matrix Gla protein (MGP), osteopontin, and bone morphogenetic protein are also present in atherosclerotic arteries. Thus, the vascular microenvironment possesses mechanisms similar to those in bone tissues to maintain mineral homeostasis. Both MGP and osteocalcin are known to be Gla-containing proteins. MGP is a secretory protein with widespread tissue expression, including in bone and vascular walls. MGP-knockout mice develop extensive calcification of arteries that rapidly becomes lethal, suggesting that MGP has an antimineralization role in the artery. In humans, osteocalcin is expressed parallel to MGP in both normal and atherosclerotic vessels and is also detected in human carotid arteries from endarterectomy samples. Thus, the two Gla proteins, osteocalcin and MGP, could play a pivotal role in not only bone mineralization but also vascular wall calcification [13]. However, at present, little is known about whether serum osteocalcin secreted from osteoblasts in bone or osteoblast-like cells in vessels actually could modulate atherosclerosis. Thus, further studies are needed to clarify the pathophysiological processes underlying the relationship between serum osteocalcin level and atherosclerosis parameters.

The circulating measure of total osteocalcin includes both carboxylated and uncarboxylated forms [35]. Animal and in vitro data implied that only the uncarboxylated form of osteocalcin functioned hormonally in the regulation of glucose homeostasis and energy metabolism. The latest researches based on population showed that elevated levels of both carboxylated and uncarboxylated forms of osteocalcin were associated with improved glucose tolerance; but the uncarboxylated form was related to insulin secretion, and the carboxylated form was associated with insulin resistance. Our study only measured total osteocalcin and did not have measurements of uncarboxylated osteocalcin; therefore, we could not further verify this hypothesis. It may be of interest to elucidate this potential mechanism in the context of the uncarboxylated and carboxylated forms and determine the direction of causality in a further longitudinal study.

Strengths of this study include the large size of the cohort, the use of early morning blood samples to minimise any effect of diurnal variation on TOC levels and the storage of aliquots at −80°C until assay. We excluded men who had conditions known to affect osteocalcin levels namely bone fracture, Paget’s disease and use of bisphosphonate or glucocorticoid therapy. However, As a cross-sectional study, there are several limitations. The mechanisms underlying these associations are still to be explored. The present findings are inherently limited in the ability to eliminate causal relationships between osteocalcin and atherosclerosis. Although most potential confounders were carefully controlled, since some of the study population had several risk factors including hypertension, and dyslipidemia, we could not eliminate the possible effect of underlying diseases and medications used for these diseases on the present findings. Further prospective population-based studies are needed to investigate the mechanisms in order to answer these questions.

In conclusion, our study indicated that serum osteocalcin levels were significantly associated with carotid atherosclerosis in patients with type 2 diabetes, even after adjustment for other potential confounders. This may reflect the role of osteocalcin as a circulating endocrine factor which regulates glucose metabolism and thereby cardiovascular risk in patients with type 2 diabetes. Prospective studies are needed to assess the time course and relevance of serum osteocalcin in the development of atherosclerosis in patients with type 2 diabetes.

## Competing interests

The authors declare that they have no competing interests.

## Authors’ contributions

Conceived and designed the experiments: JL. Analyzed the data: LS, WC. Contributed reagents/materials/analysis tools: LS, WC, BC, ZC, FW. Wrote the paper: LS, WC. All authors read and approved the final manuscript.
